# Toxicity of aged gasoline exhaust particles to normal and diseased airway epithelia

**DOI:** 10.1038/srep11801

**Published:** 2015-06-29

**Authors:** Lisa Künzi, Manuel Krapf, Nancy Daher, Josef Dommen, Natalie Jeannet, Sarah Schneider, Stephen Platt, Jay G. Slowik, Nathalie Baumlin, Matthias Salathe, André S. H. Prévôt, Markus Kalberer, Christof Strähl, Lutz Dümbgen, Constantinos Sioutas, Urs Baltensperger, Marianne Geiser

**Affiliations:** 1 Institute of Anatomy, University of Bern, 3012 Bern, Switzerland; 2 Laboratory of Atmospheric Chemistry, Paul Scherrer Institute (PSI), 5232 Villigen, Switzerland; 3 Department of Civil and Environmental Engineering, University of Southern California, Los Angeles, CA 90089, United States of America; 4 Division of Pulmonary, Critical Care & Sleep Medicine, Miller School of Medicine, University of Miami, Miami, FL 33136, USA; 5 Centre for Atmospheric Sciences, Department of Chemistry, University of Cambridge, Cambridge CB2 1EW, UK; 6 Department of Mathematics and Statistics, Institute of Mathematical Statistics and Actuarial Science, University of Bern, 3012 Bern, Switzerland

## Abstract

Particulate matter (PM) pollution is a leading cause of premature death, particularly in those with pre-existing lung disease. A causative link between particle properties and adverse health effects remains unestablished mainly due to complex and variable physico-chemical PM parameters. Controlled laboratory experiments are required. Generating atmospherically realistic aerosols and performing cell-exposure studies at relevant particle-doses are challenging. Here we examine gasoline-exhaust particle toxicity from a Euro-5 passenger car in a uniquely realistic exposure scenario, combining a smog chamber simulating atmospheric ageing, an aerosol enrichment system varying particle number concentration independent of particle chemistry, and an aerosol deposition chamber physiologically delivering particles on air-liquid interface (ALI) cultures reproducing normal and susceptible health status. Gasoline-exhaust is an important PM source with largely unknown health effects. We investigated acute responses of fully-differentiated normal, distressed (antibiotics-treated) normal, and cystic fibrosis human bronchial epithelia (HBE), and a proliferating, single-cell type bronchial epithelial cell-line (BEAS-2B). We show that a single, short-term exposure to realistic doses of atmospherically-aged gasoline-exhaust particles impairs epithelial key-defence mechanisms, rendering it more vulnerable to subsequent hazards. We establish dose-response curves at realistic particle-concentration levels. Significant differences between cell models suggest the use of fully-differentiated HBE is most appropriate in future toxicity studies.

Exposure to airborne particulate matter (PM) is known to be a leading cause of premature death^1^. Pre-existing lung disease is a major factor for susceptibility to adverse effects^2^,^3^,^4^. Gasoline-exhaust is an important PM source^5^ but health effects of exhausts from modern gasoline cars are largely unknown. Ambient PM may be primary, via direct emission, or secondary as e.g. secondary organic aerosol (SOA) from the oxidation of organic precursors, which comprises a substantial fraction (20–90%)^6^, even during winter haze episodes^7^. Recent studies suggest that SOA from gasoline car emissions makes up the dominant fraction of organic aerosol (OA) in the Los Angeles basin where SOA concentrations can be up to three times higher than the emitted primary organic aerosol (POA)^5^,^8^. Although atmospheric processing may significantly change the properties of particles, few studies exist on pulmonary effects of aged aerosol^9^,^10^,^11^ and only one pilot study on aged gasoline exhaust^12^. Inclusion of vulnerable subpopulations in experimental studies is essential, but ethically demanding. Thus, there is an urgent need for well controlled *in vitro* systems, which replicate realistic scenarios in terms of aerosol generation, atmospheric ageing, and deposition on the primarily targeted normal and impaired human airway surface. We studied the dose-response relationship when gasoline SOA is deposited on normal or diseased airway epithelia as well as BEAS-2B cells.

## Results

### Aerosol generation and administration to lung cells

**Figure 1:**
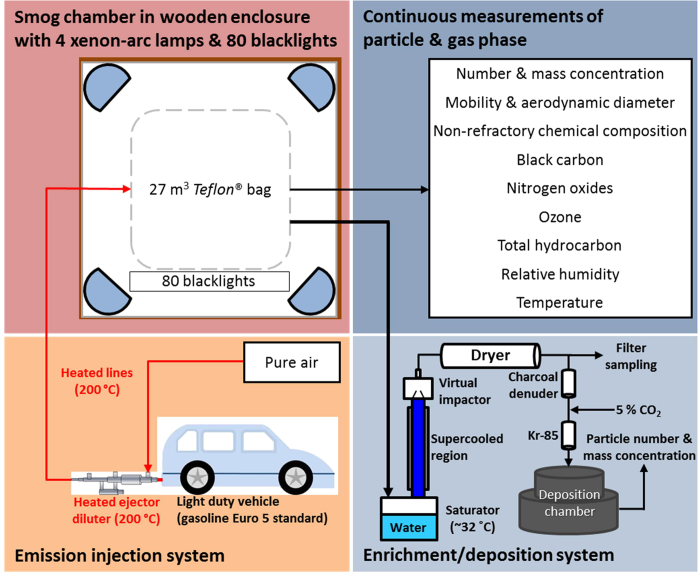
Schematic drawing of experimental set-up. The bottom left panel (light pink) illustrates the injection system. Gasoline car exhaust was diluted with pure air and directly injected via heated lines (shown in red) into the smog chamber. The upper left panel (deep pink) shows the smog chamber, including 4 xenon-arc lamps (blue semi-circles) and 80 black lights. The upper right panel (dark blue) provides an overview of the parameters measured online during the experiment. The lower right panel (light blue) represents the particle enrichment system and the aerosol deposition chamber.

**Figure 2:**
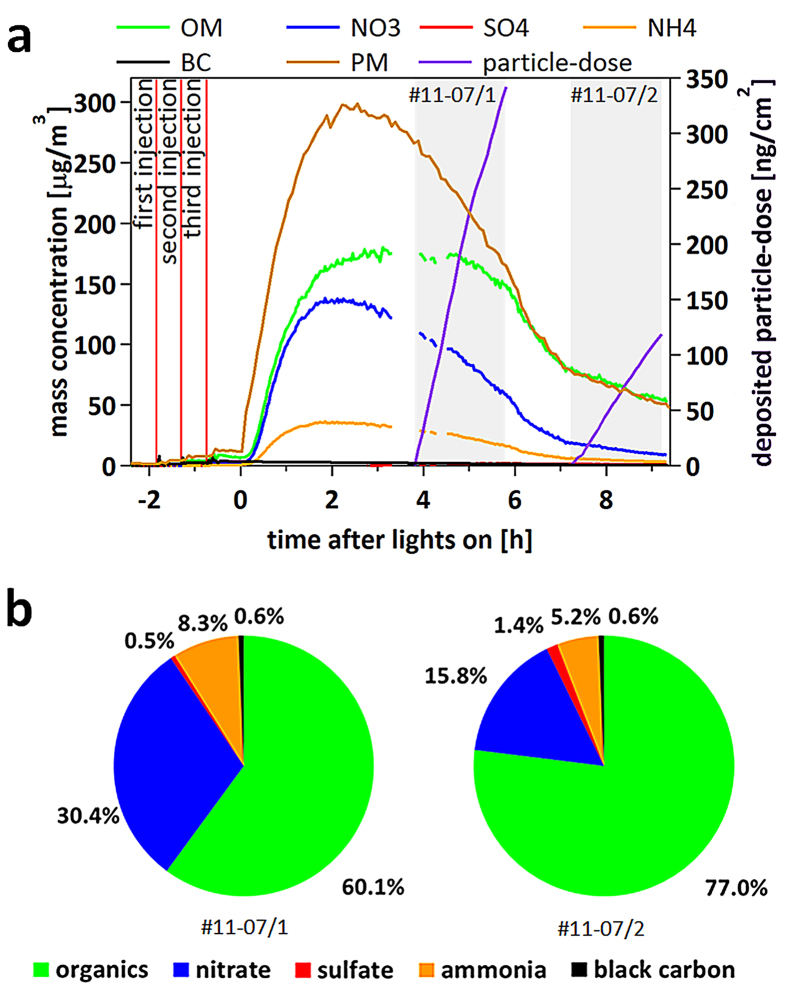
Evolution of smog chamber experiments and chemical composition of aerosol. (**a**) Non wall loss corrected evolution of particulate matter (PM) and its composition in smog chamber experiment November 07 (all other experiments are shown in the Supplementary Fig. S1). The brown line indicates total particle mass concentration measured by an SMPS (density corrected: 0.4 g cm^−3^ for primary, non-spherical particles and 1.3 g cm^−3^ for the atmospherically aged, spherical particles), while organic matter (OM), nitrate (NO_3_^−^), sulfate (SO_4_^2−^) and ammonium (NH_4_^+^) were obtained from AMS and black carbon from aethalometer measurements. The purple line illustrates the deposited particle-dose per cell surface area of each insert. The experiment begins with three successive injections into the smog chamber of 2 × 2 and 1 × 4 minutes (red vertical lines), with 30 minutes waiting time between injections, the first one resulting from a cold start of the engine. After lights are switched on, secondary aerosol (SO_4_^2−^, NO_3_^−^, OM) is formed. Grey shaded areas show the time of actual cell exposure. (**b**) Mean chemical particle composition after photochemical processing during the two hours of cell exposure derived from AMS and aethalometer measurements.

Gasoline exhaust was sampled during repeated engine starts with short idling periods via a heated inlet system, yielding total dilution ~1000, including the clean smog chamber air (see Fig. 1 for experimental set-up). UV lights were used to simulate atmospheric photochemistry in the chamber after the chemical composition of the fresh, primary emissions had been characterised. Figure 2 shows the composition of gasoline exhaust PM in the smog chamber and its evolution during ageing. Primary emissions consisted of a mix of components (on average 57% black carbon [BC] and 43% POA, number mode diameter 80 nm). Ageing produced as much as 45–180 μg m^−3^ of SOA (without wall loss correction) and the mode diameter increased to 250 nm (Fig. 2 and Supplementary Fig. S1). PM concentrations of 20–100 μg m^−3^ are typically encountered in urban areas throughout Europe and the US but may reach several 100 μg m^−3^ in megacities^13^,^14^,^15^. SOA from the smog chamber was enriched upon reaching its maximum mass concentration by a factor of 6–30 with a Versatile Aerosol Concentration Enrichment System (VACES)^16^ and fed to the online aerosol deposition chamber to expose cell cultures continuously for two hours (Fig. 1). The gas phase was removed from the sample air before entering the aerosol deposition chamber by a charcoal denuder to ensure that the cell response was exclusively due to particles. The particle dose deposited on the cells from nine exposures was in the range of about 10 to 350 ng cm^−2^, approximating the expected daily doses to the human tracheobronchial area obtained at an ambient pollution level of 20 to 1000 μg m^3^ PM (for calculations please see Supplementary Table S1). For perspective, US air quality standards require the 3-yr averages of the 98^th^ percentile of 24-h PM_2.5_ concentrations (particles with aerodynamic diameter ≤2.5 μm) to not exceed 35 μg m^−3^. The European Union has set a daily limit value of 50 μg m^−3^ for PM_10_ (particles with aerodynamic diameter ≤10 μm)^17^.

PM composition was dominated by organics during all exposures (Fig. 2b and Supplementary Fig. S1). BC contributed 0.6–3.6% to total PM, and ammonium and sulfate 1.6–8.3% and 0–1.5%, respectively. The two largest mass fractions were nitrate ranging between 4.7–30.4% and organic matter (OM, almost entirely SOA) between 60.1–93.6%. Teflon filter analysis revealed mass loadings of BC, water-soluble organic carbon (WSOC), redox active transition metals (Fe, Cu, Mn, Ni), as well as n-alkanes deposited in the aerosol deposition chamber to linearly increase with particle dose (*R*^*2*^ = 0.60–0.98) (Supplementary Fig. S2), thus confirming consistent chemical composition of the different samples across experiments.

### Cellular phenotypes and responses to aerosol exposure

**Figure 3:**
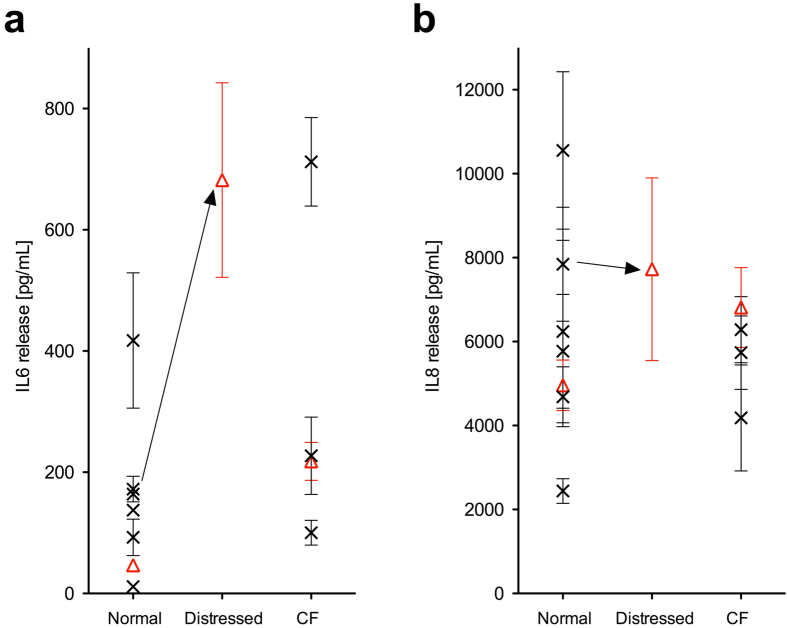
Baseline cytokine release in fully differentiated HBE cells from various normal and CF donors. Basolateral release of (**a**) interleukin (IL)-6 and (**b**) IL-8 from untreated HBE cell cultures of 7 normal and 4 CF donors into the culture medium during 24 h, presented as mean of triplicate cell cultures ± standard error (s.e.m.) for each donor. The 3 donors used in the present study are shown as red triangles. Distressing donor cells by antibiotics treatment during differentiation resulted in increased baseline release of IL-6 but not IL-8. HBE cells: human bronchial epithelial cells; CF: cystic fibrosis.

Triplicate cultures of four cell models at air-liquid interfaces (ALI) were exposed simultaneously to the aerosol of the atmospherically aged gasoline exhaust particles: two fully differentiated human bronchial epithelia (HBE) derived from normal donors, one HBE from a cystic fibrosis (CF) donor, and the proliferating human bronchial epithelial cell line BEAS-2B. HBE cells from one normal donor were treated with antibiotics during the early re-differentiation phase to simulate distressed airway epithelia. HBE cells used for exposure experiments complied with the pre-set conditions for the phenotype of the respective cell model (normal and CF HBE, for details see methods section), with regard to structure and function as a respiratory epithelium. In addition, the baseline cytokine profile, of normal and CF HBE assessed by quantifying the release of IL-6 and IL-8 during 24 h into the basal medium was comparable to that of HBE from other normal (n = 6) and CF (n = 3) donors (Fig. 3 and Methods). Particles were deposited on the apical cell surface under physiological conditions in the aerosol deposition chamber^11^,^18^. Control cultures were exposed to particle-free smog chamber air by mounting a filter inline prior to the aerosol deposition chamber. Bio-markers for pulmonary toxicity were assessed 24 h after aerosol exposure to capture acute cellular responses.

**Figure 4:**
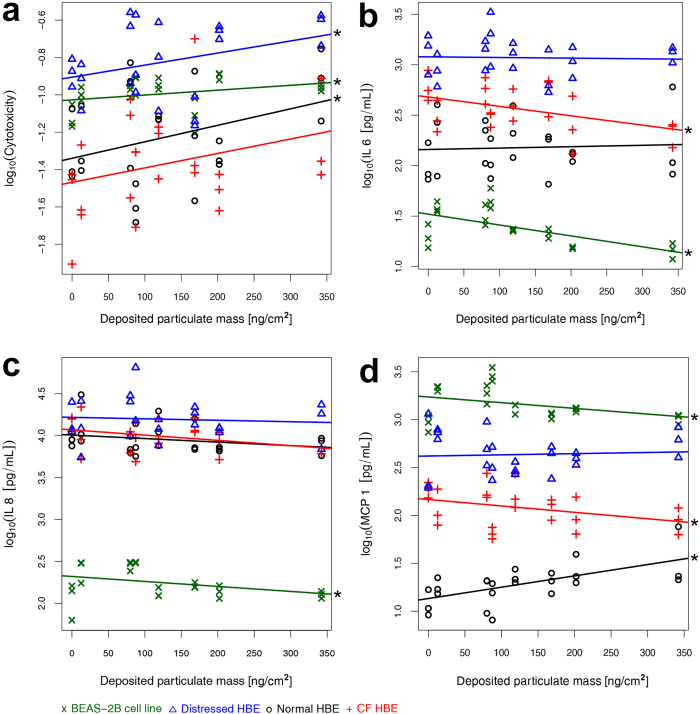
Cellular responses to increasing particle dose. (**a**) Cytotoxicity measured as fraction of total lactate dehydrogenase (LDH) released from damaged cells into the apical compartment. The inflammatory response was assessed by release of the cytokines (**b**) interleukin (IL)-6, (**c**) IL-8 and (**d**) monocyte chemotactic protein (MCP-1) . Data are presented as individual values of each cell culture. Linear trend lines for each cell model were determined using standard linear regression (see Methods section for details of statistical analyses). Stars (*) indicate a significant (p < 0.05) linear correlation to particle dose. BEAS-2B: human bronchial epithelial cell line; HBE cells: human bronchial epithelial cells; CF: cystic fibrosis.

In response to a range of deposited particle mass from 13–342 ng cm^−2^, cytotoxicity increased in all cell models on average 1.7-fold compared to p-free air exposed controls (Fig. 4a). A significant (p < 0.05) linear correlation to particle dose was confirmed in all cell models except in CF epithelia (Fig. 4a, Supplementary Table S2).

The release of interleukin (IL)-6, a classical pro-inflammatory cytokine, and IL-8, a major chemo-attractant to neutrophils, decreased significantly (p < 0.05) in BEAS-2B cells (IL-6, IL-8) and CF epithelia (IL-6), whereas no statistically relevant dose-response was measured for normal and distressed HBE (Fig. 4b,c, Supplementary Table S2). Similar to the interleukins, monocyte chemotactic protein (MCP)-1, a chemoattractant to monocytes and lymphocytes, decreased with significant correlation to particle dose in BEAS-2B cells (p = 0.0002) and CF HBE (p = 0.0328). In contrast, an increase of MCP-1 was observed in normal (p = 0.0031) and distressed HBE (not significant) (Fig. 4d, Supplementary Table S2). Tumour necrosis factor (TNF)-α, a known regulator of local and systemic inflammation, measured 6 h after aerosol exposure, was mostly below detection limits in all cell models and at all particle doses (data not shown).

To elucidate the susceptibility to adverse effects from aged gasoline particles due to host respiratory condition, we performed supplementary nonparametric statistics, which additionally consider so-called block effects, i.e. interference by conditions not related to particle exposure. These analyses corroborate that CF HBE and the cell line BEAS-2B respond significantly differently to increasing particle dose than normal and distressed HBE (Supplementary Table S3). The response of BEAS-2B cells, often used as a healthy airway epithelium proxy *in vitro*, is more similar to that of the disease model CF HBE (i.e. decreased release of IL-6 and IL-8) and exhibits less similarity with normal and distressed HBE (Table 1). Moreover, baseline values of all bio-markers tested were significantly (p < 0.05) different between the BEAS-2B cell line and HBE. An up to 100-fold lower IL-6 and IL-8 release in the cell line partly reflects the ~7 times lower cell density of simple cuboidal epithelial cell cultures compared to pseudostratified HBE. The significantly (p < 0.05) higher MCP-1 release in BEAS-2B cells, however, indicates high constitutive expression of this cytokine in BEAS-2B cells.

## Discussion

Our results provide not only experimental support for an increased susceptibility of persons with pre-existing pulmonary disorders to environmental PM exposure^2^,^3^,^4^, but also identify atmospherically aged gasoline particles as a causative agent. As expected, particle exposure had no disastrous consequences for the airway epithelia like exfoliation, since experimental conditions corresponded to a deposited dose in human airways during 24 hours of exposure at realistic ambient loadings. However, the increased cell death observed renders the epithelia more vulnerable to damage via subsequent exposure to anthropogenic or biogenic air constituents. Moreover, the decreased cytokine release as observed for CF epithelia (and BEAS-2B cells) weakens the target tissue’s capacity to adequately respond to such an insult. A further consequence of injured tissue and a restrained acute response capacity is an increased risk of pulmonary infection. This is particularly detrimental for persons with pre-existing conditions.

The results of the present study are in line with our previous data from *in vitro* exposure of primary human airway epithelia to atmospherically aged aerosols of diesel and wood combustion^10^. This previous study^10^ was performed at lower particle doses (1.3–7.7 ng cm^−2^) but similar experimental conditions (without a VACES) and indicated similar pulmonary toxicity of SOA from combustion of diesel and from biomass burning. In addition, our data support epidemiologic findings that adverse health effects occur even at minimal particle doses^1^,^19^, indicating the absence of any “no observed adverse effect level” (NOAEL).

Cellular responses also depend on how particles are deposited onto the cells and at which dose^11^. This likely explains differences between our findings and some other *in vitro* studies^e.g.,^^20^,^21^. Most *in vitro* studies are greatly over-dosed compared to the levels of realistic inhalation exposure and significantly deviate from realistic modes of particle application and target tissue^22^; caution should therefore be used in interpreting some of these results. Realistic exposure simulations require atmospherically relevant mass, number concentration and composition of atmospheric particles and, critically, incorporation of atmospheric ageing.

These combined smog chamber/cell exposure experiments provide the most realistic representation of the ambient situation in the laboratory, incorporating photochemical ageing of the aerosol, particle enrichment without affecting chemical and physical particle properties, and deposition of particles directly out of a continuous air flow onto cell cultures from human donors under physiological conditions. Finally, the use of re-differentiated airway epithelia seems indispensable, since (i) the response of the simplistic cell line model BEAS-2B deviates significantly from that of normal HBE and (ii) the realistic human cell model allows including susceptible subgroups of the population in experimental studies otherwise ethically not possible. It has to be emphasized, however, that inter-cell culture and inter-donor variability need to be taken into special consideration when using HBE cells.

The *in vitro* study presented here clearly shows that even a single, short-term exposure to atmospherically aged gasoline exhaust particles increases necrotic cell death in a dose-dependent manner in normal and in compromised respiratory epithelia and decreases cytokine release in CF epithelia. Aged gasoline exhaust particles thus render airway epithelia more vulnerable to secondary exposure to air pollutants.

## Methods

### Summary of experimental procedure

Three separate start-up and idle (2–4 minutes idling, several minutes apart) emissions from a Euro 5 gasoline car were sampled via heated lines into a 27 m^3^ smog chamber^23^. After equilibration, nitrous acid (an OH radical precursor) was continuously injected and UV lights were switched on initiating SOA formation. Particle composition including black carbon, non-refractory PM and size distributions were monitored by an aethalometer (AE33, Magee Scientific Corp., Berkeley, CA, USA), a high resolution time-of-flight aerosol mass spectrometer (HR-ToF-AMS, Aerodyne Research Inc., Billerica MA, USA) and a scanning mobility particle sizer (SMPS, custom built) respectively. Maximum SOA concentrations were achieved after ~4 h of ageing, at which point the chamber aerosol was sampled through a VACES, where it was enriched up to 30 times^24^. The enriched aerosol was split into two streams. One was directed through a Teflon filter for measurement of BC, WSOC, metals and speciated organics^9^, the second directed through a charcoal denuder to remove gases prior to deposition onto airway epithelia at ALI^11^,^25^ in an online aerosol deposition chamber^18^. Relevant pulmonary toxicity bio-markers were measured 24 h after exposure^11^. Cytotoxicity was assessed by apical lactate dehydrogenase (LDH) release from the cytosol of damaged cells. Inflammatory responses were assessed by quantifying basolateral release of interleukin (IL)-6, IL-8, monocyte chemotactic protein (MCP)-1 and tumour necrosis factor (TNF)-α.

#### Cell cultures

Re-differentiated HBE and BEAS-2B cells were prepared according to standard protocols of our laboratory^11^. In short, HBE cells were isolated^25^ from two normal and a CF (homozygous for the ΔF508 mutation) human organ donor. Normal lungs deemed not suitable for transplant were obtained from the Life Alliance Organ Recovery Agency (LAORA) of the University of Miami, Miami, FL, USA. Institutional Review Board (IRB)-approved consent for research with these tissues was obtained by LAORA and conformed to the declaration of Helsinki. CF cells donated by a transplant recipient were collected according to IRB approved protocols. HBE cells were grown and differentiated at a permanent ALI on microporous 24-mm Transwell® inserts (polyester membrane, 0.4 μm pores; Corning, Vitaris, Baar, Switzerland) coated with human placental collagen Type IV (Sigma-Aldrich, Buchs, Switzerland)^25^,^26^. Basal medium was changed daily and mucus removed by washing cell surfaces with Dulbecco’s phosphate-buffered saline (DPBS, pH 7.4) every other day. Differentiation was assessed by the presence of beating cilia and visible mucus secretion typically after 21 days of culture at ALI. Cells from one normal donor were treated with ciprofloxacin (10 μg mL^−1^; Sigma) for 4 days at the beginning of *in vitro* re-differentiation resulting in distressed airway epithelia.

To account for inter-cell-culture and inter-donor variability, HBE cultures used for exposure experiments were evaluated as follows: (i) (ultra)structure and function as previously described^27^ confirming microscopically and visually the presence of a pseudostratified epithelium containing ciliated, mucus and basal cells, junctional complexes, ciliary beating, and mucus production (sticky mucus in CF HBE); (ii) baseline cytokine profiles by quantifying 24-h release of IL-6 and IL-8 into the basal medium by untreated HBE cells and subsequent comparison of data to those of fully differentiated and functional HBE cells from other normal (n = 6) and CF (n = 3) donors. The results of this analysis demonstrate that baseline release of IL-6 and IL-8 by normal and CF HBE cells used in the present study were comparable to the values obtained from HBE cells of other donors with equivalent health status (Fig. 3). The data also show that distressing normal HBE cells by antibiotics treatment during differentiation resulted in a substantial 4.0-fold increase in baseline release of IL-6 but not IL-8.

Submersed BEAS-2B cultures were grown on microporous 24-mm Falcon^®^ inserts (polyester membrane, 0.4 μm pores; Becton Dickinson AG, Milian, Geneva, Switzerland). For aerosol exposure of BEAS-2B cultures apical medium was reduced to a minimum (<1 mm in height) to mimic ALI conditions.

#### Aerosol generation and processing

The smog chamber consists of a 27 m^3^ fluorinated ethylene propylene bag suspended in a temperature-controlled wooden enclosure^23^. Four xenon-arc lamps and 80 black lights simulate the solar radiation spectrum. Temperature and relative humidity were recorded throughout the experiments. A Euro 5 gasoline light duty vehicle (TypE) served as source of particles and gaseous precursors. The exhaust of 3 separate start-up and idle (2–4 minutes idling, several minutes apart) was sampled through two Dekati^®^ ejector diluters in parallel. The sampled exhaust was diluted by a factor of 12 (3 L min^−3^ sample flow and 36 L min^−3^ dilution air) and transferred to the chamber by heated (200 °C) injection lines, preventing condensation of semi-volatile organic compounds. After the injections, convective turbulences due to a small vertical temperature gradient led to a homogeneous mixing of the pollutants within 30 min. OH radicals were then produced by photolysis of nitrous acid, which was continuously added (2.5 L min^−1^) to the chamber 30 min prior and throughout the ageing process. After about 4 h of photochemical ageing, SOA was sampled, enriched and deposited on respiratory epithelia.

#### Aerosol enrichment

A two-stage VACES^16^ was used to provide concentration-enriched particles to the deposition chamber^18^. The atmospherically processed aerosol was first drawn through a saturator–condenser system which grows particles to 3–4 μm aqueous droplets. Grown particles were hereafter concentrated by virtual impaction, and then directed through a diffusion dryer to bring them back to their original size. The diffusion-dried stream was subsequently split into two flows, each directed to a 37-mm Teflon filter or the aerosol deposition chamber. The enrichment factor was varied by changing the total-to-minor flow rates of the virtual impactor. By varying the enrichment by up to a factor of 30, the dose of deposited particles was varied accordingly allowing study of the dose-response relationship.

#### Cell exposures

In our aerosol deposition chamber, the aerosol was applied to the airway epithelia for 2 h directly out of a conditioned air-flow closely mimicking physiological conditions. This chamber is described in detail elsewhere^18^, therefore, only a short description is given here. After the VACES, the air stream passes a charcoal denuder to remove pollutant gaseous species and a Kr-85 source to establish particle-charge equilibrium. The particles are then sucked into the aerosol deposition chamber at a flow rate of 50 mL min^−1^ per cell culture for up to 12 cell cultures simultaneously. Before particles are deposited onto the cell cultures, the aerosol flow is conditioned to physiological conditions in the lung, i.e., 37 °C and >90% relative humidity. Particles are deposited out of the continuous airflow using an alternating electric field of 2 kV cm^−1^. This results in an efficient deposition of about 8% of all particles in the sub-micrometer size range. Particle deposition was shown to be uniform over the entire cell culture assuring even exposure of the cells and reproducible biological results. As previously published, three different measurements of particle mass concentrations were used to determine the fraction of the particles deposited onto cell cultures: (i) bypassing the cell chamber; (ii) through the cell chamber with and (iii) without electric field. Retrieved deposition efficiency is in good agreement with previously published data^18^. Thereafter, particulate mass deposited on cell cultures was determined using only the SMPS measurements downstream of the aerosol deposition chamber.

#### Aerosol and gas phase characterisation

A high-resolution time-of-flight aerosol mass spectrometer (HR-ToF-AMS, Aerodyne Research Inc., Billerica MA, USA) delivered highly time- and size-resolved online measurements of particle composition. Working principles and data analysis protocols can be found elsewhere^28^. The HR-ToF-AMS provides quantitative mass spectra of non-refractory PM_1_ components including organic aerosol, ammonium, nitrate, and sulfate^28^. Time-resolved measurements of black carbon (BC) were obtained using an aethalometer (AE33, Magee Scientific, Berkeley, CA, USA)^29^. This instrument measures attenuation on two spots from different parallel sampling flows resulting in different filter loadings. Hence, the “loading effect” is online compensated. Two scanning mobility particle sizers (SMPS) were connected directly to the smog chamber and downstream of the aerosol deposition chamber, respectively. To determine mass concentrations from the SMPS measurements, particle density was estimated from the combination of mobility (SMPS) and aerosol mass collected and weighed on Teflon filters.

Filter samples collected during cell exposure (n = 6) were analysed for BC (Magee Scientific Model OT-21 SootScan™ Optical Transmissometer, Berkeley, CA, USA), WSOC, total metals and elements as well as speciated organics, including polycyclic aromatic hydrocarbons, hopanes, n-alkanes and organic acid^9^. With the exception of n-alkanes and organic acids, most organics exhibited a negligible concentration. BC measurement was conducted on the individual filter samples. On the other hand, due to insufficient mass for chemical analysis, filters collected from exposures with similar concentration enrichment factor were pooled together and analysed as composited samples. For WSOC and metals analysis, filters collected from exposures 10/29 and 11-07/2 were analysed as one composited sample. Similarly, filters collected from exposures 11-05 and 11-02/2 were analysed as a single composited sample, while filters collected from exposures 10/31 and 11-07/1 were each individually analysed. For organics measurement, Filters collected from exposures 11-05 and 11-02/2 were composited while the sample collected on 11-07/1 was directly analysed. In the gas phase, NO, NO_2_ and NO_x_ (Trace level 42C, Thermo Environmental Instruments with a photolytic converter and 9841A Monitor Labs NO_x_ analyser), O_3_ (Environics S300 ozone analyser) and total hydrocarbons (THC, J.U.M. Model VE 7 THC analyser with flame ionization detector) were measured.

#### Bio-markers

Cytotoxicity was measured by release of LDH from the cytosol of damaged cells. Apical washes (DPBS supplemented with 0.5% bovine serum albumin) of the primary cultures and apical media of the BEAS-2B cells were collected 24 h after aerosol exposure and analysed using the colorimetric cytotoxicity detection kit^PLUS^ (Roche Diagnostics AG, Rotkreuz, Switzerland) according to the manufacturer’s instructions. Apical LDH release was normalized to maximal releasable LDH. Release of the inflammatory mediators IL-6, IL-8 and MCP-1 into the basal media was assessed 24 h after aerosol exposure. Samples were collected and stored at −20 °C. Cytokines were analysed using a Bio-Plex bead-based suspension array system and appropriate detection kits (Bio-Rad Laboratories, Reinach, Switzerland) according to the manufacturer’s protocol.

#### Statistics

Cell cultures were exposed to each particle dose in triplicates. Data for cytotoxicity and cytokine release are presented as individual values and the corresponding linear trend line including the p-free control and all particle doses. Results were considered as statistically significant for p < 0.05. Raw data *Y*_raw_ were replaced with their log-transformed values *Y* = log_10_(*Y*_*raw*_) for statistical analyses. Four different cell models *I* = 1,2,3,4 were considered, where *I* = 1 stands for normal HBE. For each cell model *i* there are measurements of a certain response variable *Y* under eight different treatments *j* = 1,2,…,8, where *j* = 1 corresponds to the p-free control experiment, and *j* = 2,3,…,8 correspond to exposure to increasing doses of gasoline combustion particles. Additionally, for each response measurement there are repetitions *k* = 1,2,…,*K*_*ij*_. Preliminary analyses indicated that modelling the *Y*_*ijk*_ as independent random variables with distribution *Y*_*ijk*_ ~ *N(a*_*ij*_*, σ*^*2*^_*i*_) is plausible, where *a*_*ij*_ is a mean response depending on cell model and treatment, and *σ*_*i*_ > 0 is the standard deviation depending on the cell model and reflects measurement errors and the natural variability of cell cultures. For any specific cell model *i*, we investigated whether there is a significantly positive or negative linear trend in the eight levels *a*_*i*1_*, a*_*i*2_*, …. a*_*i*8_. Precisely, we estimated the trend parameter *Ψ*_*i*_ of the corresponding regression line and its standard error. By means of standard methods for linear models^30^ we obtained a *p*-value for the null hypothesis that *Ψ*_*i*_ equals 0 and a 95%-confidence interval for *Ψ*_*i*_.

To test whether there is a significant difference between two different cell models across all particle doses Mann-Whitney^31^ statistics was performed with stratification for different days of the experiment and different treatments to avoid so-called block effects.

## Additional Information

**How to cite this article**: Künzi, L. *et al*. Toxicity of aged gasoline exhaust particles to normal and diseased airway epithelia. *Sci. Rep*. **5**, 11801; doi: 10.1038/srep11801 (2015).

## Supplementary Material

Supplementary Information

## Figures and Tables

**Table 1:**
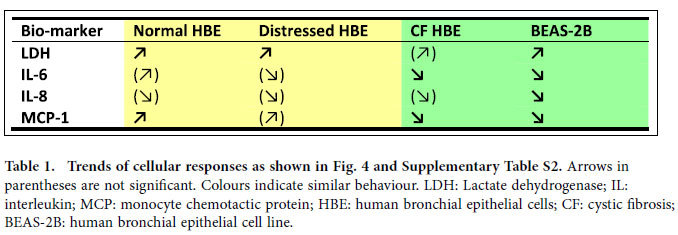
Trends of cellular responses as shown in Fig. 4 and Supplementary Table S2.
