# Supplementary material for: Toxicity of aged gasoline exhaust particles to normal and diseased airway epithelia
Source: Sci Rep. 2015 Jun 29;5:11801. doi: 10.1038/srep11801 (PMC4484354; doi:10.1038/srep11801)
Supplement: Supplementary Information [file srep11801-s1.pdf]

Toxicity of aged gasoline exhaust particles to normal and diseased airway epithelia

Lisa Künzi, Manuel Krapf, Nancy Daher, Josef Dommen, Natalie Jeannet, Sarah Schneider, Stephen Platt, Jay G. Slowik , Nathalie Baumlin, Matthias Salathe, André S.H. Prévôt, Markus Kalberer, Christof Strähl, Lutz Dümbgen, Constantinos Sioutas, Urs Baltensperger, Marianne Geiser

Supplementary Information

Supplementary Figures

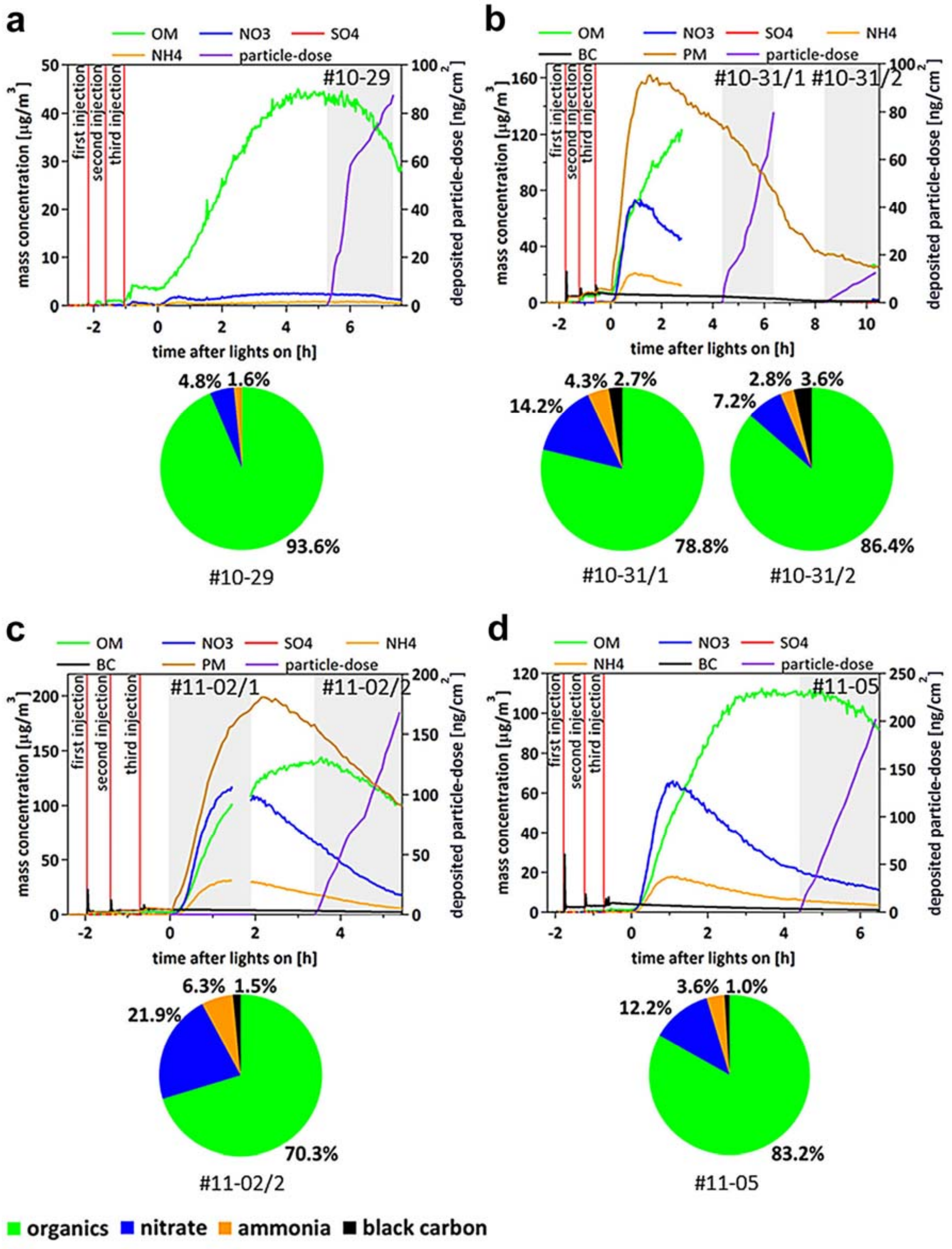

Supplementary Figure S1 (a-d). Evolution of smog chamber experiments and chemical composition of aerosol. Top panel: Non wall loss corrected evolution of particulate matter (PM) and its composition in all smog chamber experiments (except for November 07, which is shown in Fig. 2 in the main manuscript). Brown lines indicate total particle mass concentration measured by an SMPS (density corrected: 0.4 g cm^{-3} for primary, non-spherical particles and 1.3 g cm^{-3} for the atmospherically aged, spherical particles); organic matter (OM), nitrate (NO_3^-), sulfate (SO_4^{2-}) and ammonium (NH_4^+) were obtained from AMS and black carbon from aethalometer measurements. The purple line illustrates the deposited particle-dose per cell surface in each insert. The experiments started with three successive injections into the smog chamber of 2 × 2 and 1 × 4 minutes each (red vertical lines), with 30 minutes waiting time between injections, the first one resulting from a cold start of the engine. After lights are switched on secondary aerosol (SO_4^{2-}, NO_3^-, OM) is formed. Grey shaded areas show the time of actual cell exposure. **Bottom panel**: Mean chemical particle composition during the two hours of cell exposure derived from AMS and aethalometer measurements.

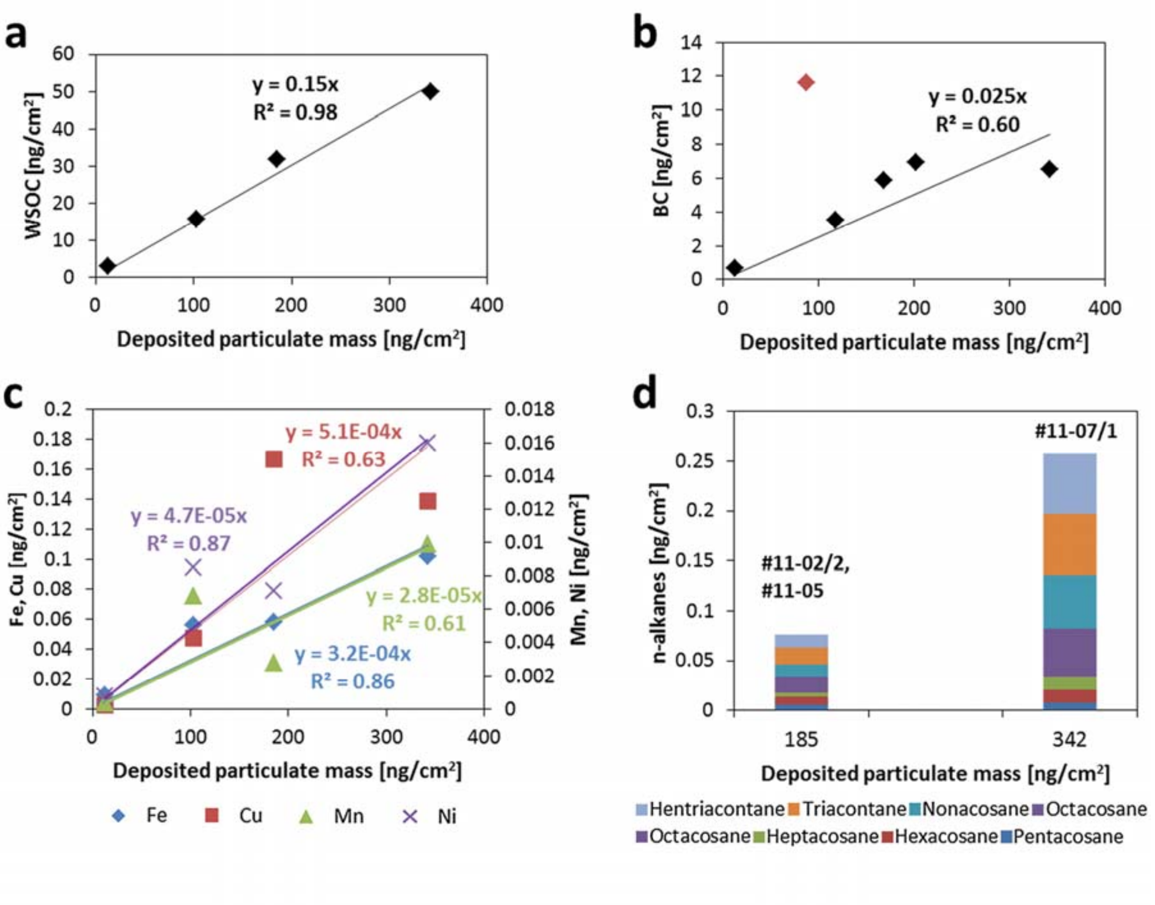

Supplementary Figure S2. Chemical properties of the deposited aerosol. Chemical composition of the aerosol was comparable over the individual exposure experiments as indicated by the good correlation between the total aerosol mass deposited per cm^2 cell culture insert and the deposited concentration of **(a)** water-soluble organic carbon (WSOC), **(b)** black carbon (BC), **(c)** Fe, Cu, Mn, Ni and **(d)** n-alkanes. Trend lines were obtained from linear regression. The data point shown in red in **(b)** represents a potential outlier that was excluded from the regression analysis.

Supplementary Tables

Supplementary Table S1. Calculation of deposited particle dose in human tracheobronchial (TB) tract at various ambient PM concentrations

		Particle size, diameter [nm]	
		**100**	**250**
**Particle density** [g /cm^3]		1.00	1.00
**Tidal Volume**, V_T [m^3][1]		0.000625	0.000625
**Beathing frequency**, *f* [min^{-1}][a,b]		12	12
**Inhaled air volume/h** (adult) [m^3][a,b]		0.45	0.45
**Inhaled air volume/24h** (adult) [m^3]		10.8	10.8
**Surface area TB tract** [cm^2][c]		2471	2471
**Deposition efficiency**[a,b2]		0.111	0.066
**Mass conc.** [µg/m^3]	20		
**Mass/surface area TB/24h** [ng/cm^2]		9.7	5.8
**Mass conc.** [µg/m^3]	100		
**Mass/surface area TB/24h** [ng/cm^2]		49	29
**Mass conc.** [µg/m^3]	500		
**Mass/surface area TB/24h** [ng/cm^2]		243	144
**Mass conc.** [µg/m^3]	1000		
**Mass/surface area TB/24h** [ng/cm^2]		485	288

a Anjilvel, S. & Asgharian, B. A multiple-path model of particle deposition in the rat lung. *Fundam. Appl. Toxicol.* **28**, 41-50 (1995).

b National Institute for Public Health and the Environment (RIVM). Multiple Path Particle Dosimetry Model (MPPD v 1.0): A Model for Human and Rat Airway Particle Dosimetry. Bilthoven, The Netherlands. RIVA Report 650010030 (2002).

c Mercer, R. R., Russell, M. L., Roggli, V. L., & Crapo, J. D. Cell number and distribution in human and rat airways. *Am. J. Respir. Cell Mol. Biol.* **10**, 613–624 (1994).

Supplementary Table S2. Statistical analysis of dose-response relationship - linear trend over all particle doses

Bio-marker and cell model	Estimated parameter (standard error)	p-value of "no trend" (95% conf. interval for parameter)
**Cytotoxicity**		
Normal HBE	0.0009 (0.0004)	**0.0443*** (0, 0.0017)
Distressed HBE	0.0006 (0.0003)	**0.0446*** (0, 0.0013)
CF HBE	0.0008 (0.0005)	0.1426 (-0.0003, 0.0018)
BEAS-2B	0.0003 (0.0001)	**0.0171*** (0.0001, 0.0005)
**IL-6**		
Normal HBE	0.0001 (0.0005)	0.8047 (-0.001, 0.0013)
Distressed HBE	-0.0001 (0.0004)	0.8582 (-0.0008, 0.0007)
CF HBE	-0.0009 (0.0003)	**0.0139*** (-0.0016, -0.0002)
BEAS-2B	-0.0011 (0.0001)	**< 0.0001*** (-0.0014, -0.0008)
**IL-8**		
Normal HBE	-0.0004 (0.0003)	0.2345 (-0.0011, 0.0003)
Distressed HBE	-0.0002 (0.0005)	0.7213 (-00012, 0.0008)
CF HBE	-0.0006 (0.0003)	0.0854 (-0.0014, 0.0001)
BEAS-2B	-0.0006 (0.0002)	**0.0089*** (-0.001, -0.0002)
**MCP-1**		
Normal HBE	0.0012 (0.0003)	**0.0031*** (0.0005, 0.0019)
Distressed HBE	0.0001(0.0004)	0.7659 (-0.0007, 0.001)
CF HBE	-0.0007 (0.0003)	**0.0328*** (-0.0013, -0.0001)
BEAS-2B	-0.0006 (0.0001)	**0.0002*** (-0.0009, -0.0003)

The estimated parameter is the slope of a regression line through the dose-response pairs. It quantifies the linear trend in the dose-response relationship. The p-value tests the null hypothesis that there is no linear trend at all.

Supplementary Table S3. Comparison of cell models over all particle doses

Bio-marker and cell model	Distressed HBE	CF HBE	BEAS-2B
	U_{norm} (p-value)	U_{norm} (p-value)	U_{norm} (p-value)
**Cytotoxicity**			
Normal HBE	0.0833 (< **0.0001***)	0.7639 (**0.0036***)	0.2361 (**0.0036***)
Distressed		0.9583 (< **0.0001***)	0.8333 (**0.0002***)
CF HBE			0.0833 (< **0.0001***)
**IL-6**			
Normal HBE	0 (< **0.0001***)	0.125 (< **0.0001***)	1 (< **0.0001***)
Distressed HBE		0.9306 (< **0.0001***)	1 (< **0.0001***)
CF HBE			1 (< **0.0001***)
**IL-8**			
Normal HBE	0.1884 (**0.0006***)	0.3889 (0.2493)	1 (< **0.0001***)
Distressed HBE		0.7391 (**0.0101***)	1 (< **0.0001***)
CF HBE			1 (< **0.0001***)
**MCP-1**			
Normal HBE	0 (< **0.0001***)	0.0139 (< **0.0001***)	0 (< **0.0001***)
Distressed HBE		0.9583 (< **0.0001***)	0.0417 (< **0.0001***)
CF HBE			0 (< **0.0001***)

For the value U_{norm} we considered all pairs of response measurements under comparable conditions, the first one for the cell model specified in the row and the second one for the cell model specified in the column. The number U_{norm} is the proportion of all such pairs in which the first value is larger than the second one. The p-value tests the null hypothesis that there is no systematic difference between the two cell models, i.e. that $U_{norm} = 0.5$ on average. A small p-value means that the observed deviation of U_{norm} from 0.5 is significant.
